# Supplementary material for: Association between oral microbiome and seven types of cancers in East Asian population: a two-sample Mendelian randomization analysis
Source: Front Mol Biosci. 2023 Nov 21;10:1327893. doi: 10.3389/fmolb.2023.1327893 (PMC10702768; doi:10.3389/fmolb.2023.1327893)
Supplement: Supplementary file 1 [file DataSheet1.ZIP › Supplementary Materials/MR plots for tongue/tongue═╝/Lung cancer/pheno.237_to_lung cancer_leave_one_out.pdf]

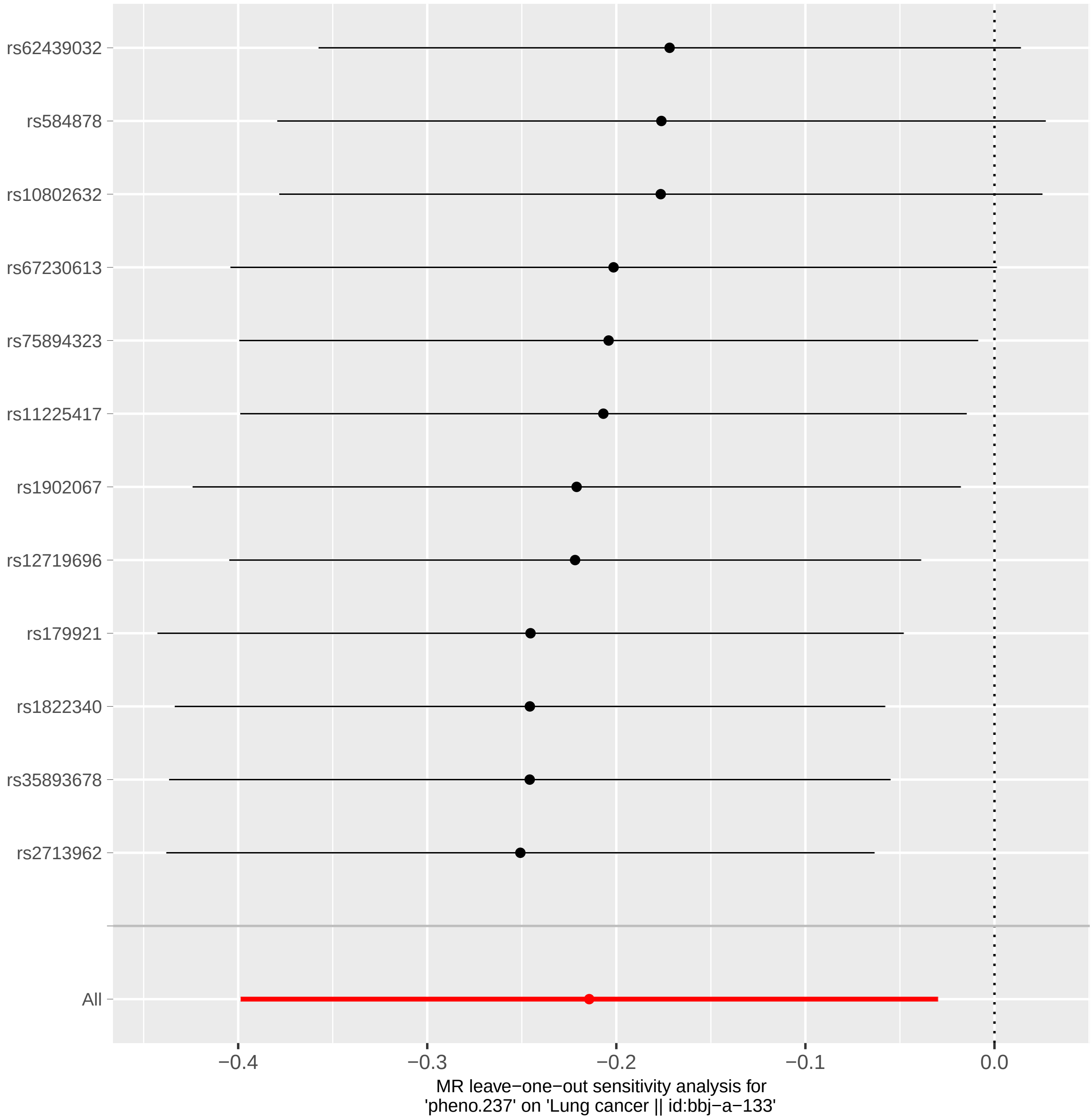

rs62439032
rs584878
rs10802632
rs67230613
rs75894323
rs11225417
rs1902067
rs12719696
rs179921
rs1822340
rs35893678
rs2713962
All
−0.4
−0.3
−0.2
−0.1
0.0
MR leave−one−out sensitivity analysis for
'pheno.237' on 'Lung cancer || id:bbj−a−133'
